# Intraoperative Specimen Ultrasonography: Is It a Reliable Tool for Margin Assessment Following Breast Conservation Surgery for Breast Carcinoma?

**DOI:** 10.7759/cureus.15806

**Published:** 2021-06-21

**Authors:** Niranjan Kumar, Megha Tandon, Chintamani Chintamani

**Affiliations:** 1 Department of Surgery, Tata Main Hospital, Jamshedpur, IND; 2 Department of General Surgery, Vardhman Mahavir Medical College and Safdarjung Hospital, Delhi, IND

**Keywords:** breast conservation surgery, intraoperative ultrasonography, frozen section, re-excision rate, lumpectomy

## Abstract

Background: Assessment of margins after breast conservation surgery is an essential part of management in breast cancer and is important in prognostication of the patient. Various intra-operative techniques like frozen section and imprint cytology are in use to ensure negative margins but have their limitations in the fact that 3D evaluation is not feasible. These lead to false negatives and also are operator dependent. In order to obviate these shortcomings, various centers are using specimen imaging (specimen mammogram and ultrasonography).

Aims and Objectives: 1) To evaluate the accuracy of specimen ultrasonography in assessing the margins following breast conservation surgery (BCS). 2) To compare the accuracy of intra-operative specimen ultra-sonography with frozen section for assessment of excision margins following BCS.

Materials and methods: Sixty-two biopsy-proven patients with breast cancer who underwent BCS were included in this prospective study at a tertiary cancer care center. The oriented specimens were evaluated by specimen ultrasonography and later by frozen section. The final histopathology served as the gold standard.

Results: Specimen ultrasonography is found to be superior to frozen section in providing detailed assessment of margins in patients undergoing breast conservation. Specimen ultrasonography was also able to detect additional lesions which might be missed on frozen section, especially the in-situ carcinoma.

## Introduction

Breast cancer is a global health problem and the leading cause of cancer-related mortality among women [1]. A multimodal approach is being followed for its management. Locally advanced breast cancer (LABC) is the most common presentation in developing countries due to illiteracy, lack of awareness, poor health care, lack of active screening and late presentation. In India, up to 30-35% of breast cancer are locally advanced at the time of presentation [2]. Breast conservation surgery (BCS) is now an acceptable option for women with early-stage breast cancer and even in some cases of locally advanced breast cancer after response to neo-adjuvant chemotherapy (NACT) [3-5]. Some patients who initially underwent breast conservation ultimately had mastectomy because of presence of close (less than 2mm) or positive surgical margins [6,7].

Obtaining negative margins is critically important for local control of breast cancer. Failure to obtain negative margins during initial surgery results in re-excision in 30-50% of patients [8]. It was observed that in various treatment strategies (BCS alone, BCS with radiation therapy, or mastectomy), negative margin status strongly correlated with long-term disease-free survival. Positive margin has a negative effect on local recurrence and disease-free and overall survival of the patient [9,10]. To minimize the current reported high re-excision rates, intraoperative assessment of margins has become increasingly desired. Intra-operative ultrasound, imprint cytology, scrape cytology and specimen mammography have all been employed [11]. Various studies have compared the sensitivity of specimen imaging with the frozen techniques in assessment of margins [11,12]. In this study, specimen ultrasonography and frozen section were both used to assess tumor margins intra-operatively. This study outlines the major challenges encountered intra-operatively to achieve negative margins and also assesses the efficacy of intra-operative specimen ultrasonography and frozen section for margin assessment compared to final histopathology (HPE).

## Materials and methods

Sixty-two patients, who underwent BCS for early breast cancer (T1-2, N0-1, and M0) in a single surgical unit during the years 2013 to 2017, were included in the study. A detailed history was recorded, triple assessment (clinical examination of both breasts, USG/mammography, core needle biopsy) of all the patients was done after taking informed consent. The patients were then staged clinically as per the TNM staging system.

In all patients, wide local excision (WLE) of the breast cancer tumor was done using the standardized technique, by palpation method (at least 1 cm margin taken around the palpable tumor) and the margins of the tumor were marked with sutures as per the standard practice. The margins were re-assessed using high-frequency ultrasound, performed immediately after the specimen was resected. Transverse and sagittal images of the resected specimen were obtained. If the margin was less than the 1 cm desired margin all around, or margins are indistinct, additional breast parenchyma was resected in that direction and sent labeled separately to pathology for evaluation. The metallic marker placed at the tumor bed after confirming the margin status and defect was closed using oncoplastic principles. If 1 cm of deep margins was not possible, pectoralis fascia is considered as appropriate margin. The margins were also assessed by frozen section. In case of positive report, the margins were excised and sent separately for histopathology marking the outer margins which were considered as the new margins.

The oriented lumpectomy specimen, oriented re-excised margins (if any) and axillary nodes were sent separately for detailed histopathological examination for confirmation of diagnosis, grade of tumor, nodal status and importantly for final histopathological margin assessment with special emphasis on the distance of the tumor from the resected margins and presence of carcinoma in situ in the margins.

Frozen section technique

**Figure 1:**
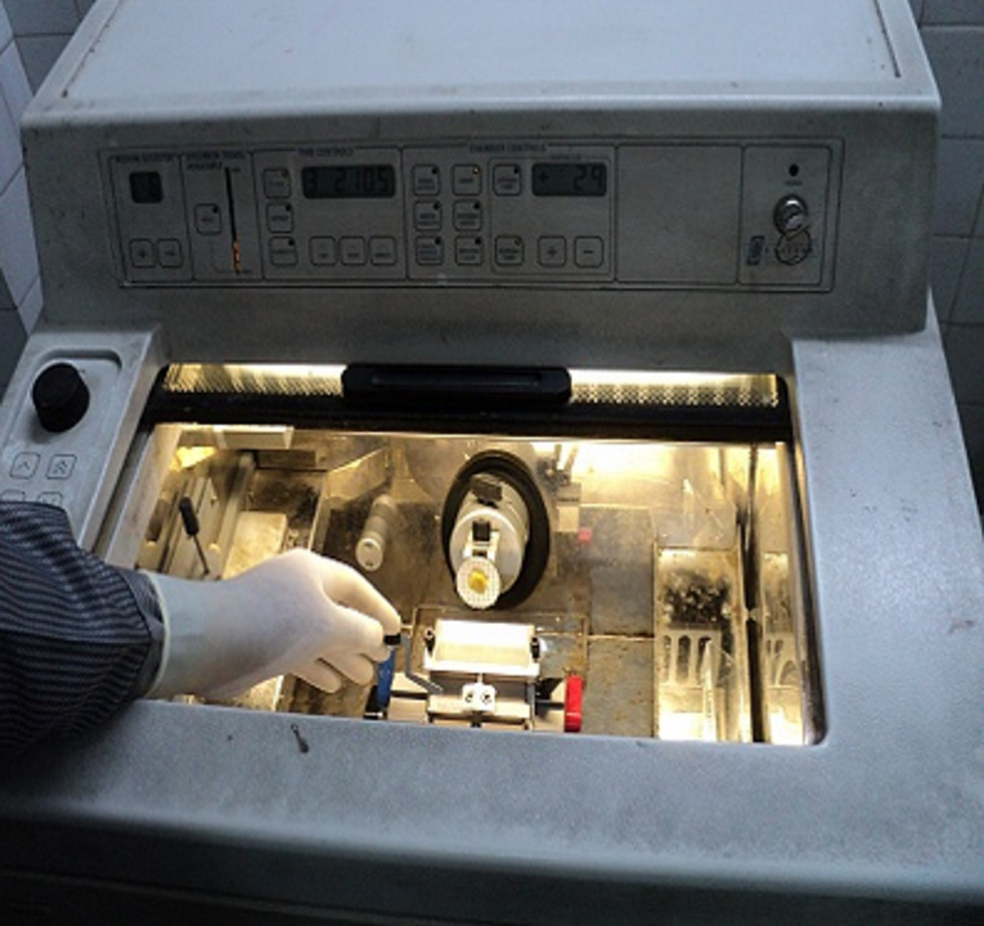
Cryostat -Tissue section cut directly on a standard cryostat

**Figure 2:**
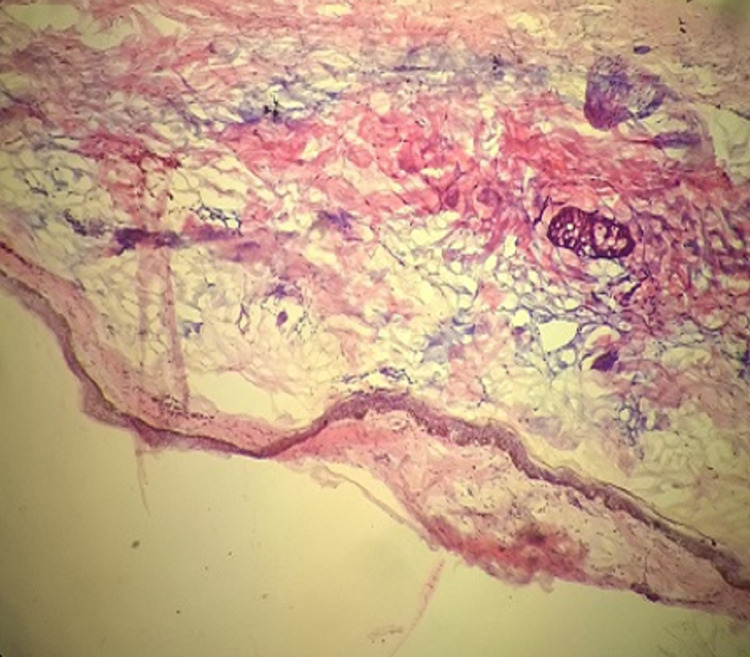
Frozen H&E stain section showing margins free from tumor: Low power view (4x) H&E: haematoxylin and eosin

The specimen was marked to record its orientation and submitted to a pathologist for the paraffin section. Frozen section biopsy (radial section) done by a standard approach involving five specimens, one from each of four margins of the specimen and one from deep surface. These specimens were evaluated for intraoperative frozen section. Frozen sections were performed by freezing the tissue in a block of specialized embedded in medium (OCT media), followed by cutting a usually 5-micron section from the block using a cryostat. The sections were adhered to glass slides, fixed in ethanol, and stained with haematoxylin and eosin (Figure 1, 2). The intra-operative frozen section analysis result for each biopsy was recorded as positive, negative, or suspicious.

Specimen ultrasonography

**Figure 3:**
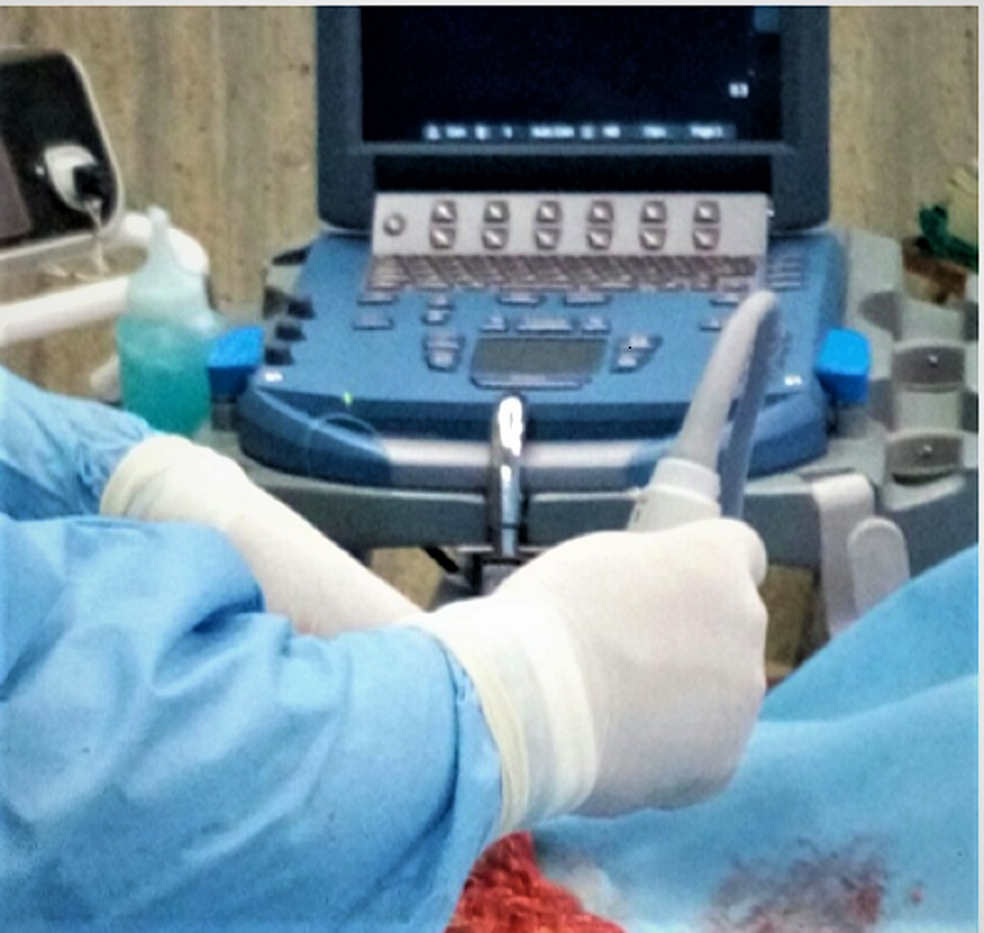
Intra-operative specimen ultrasonography.

**Figure 4:**
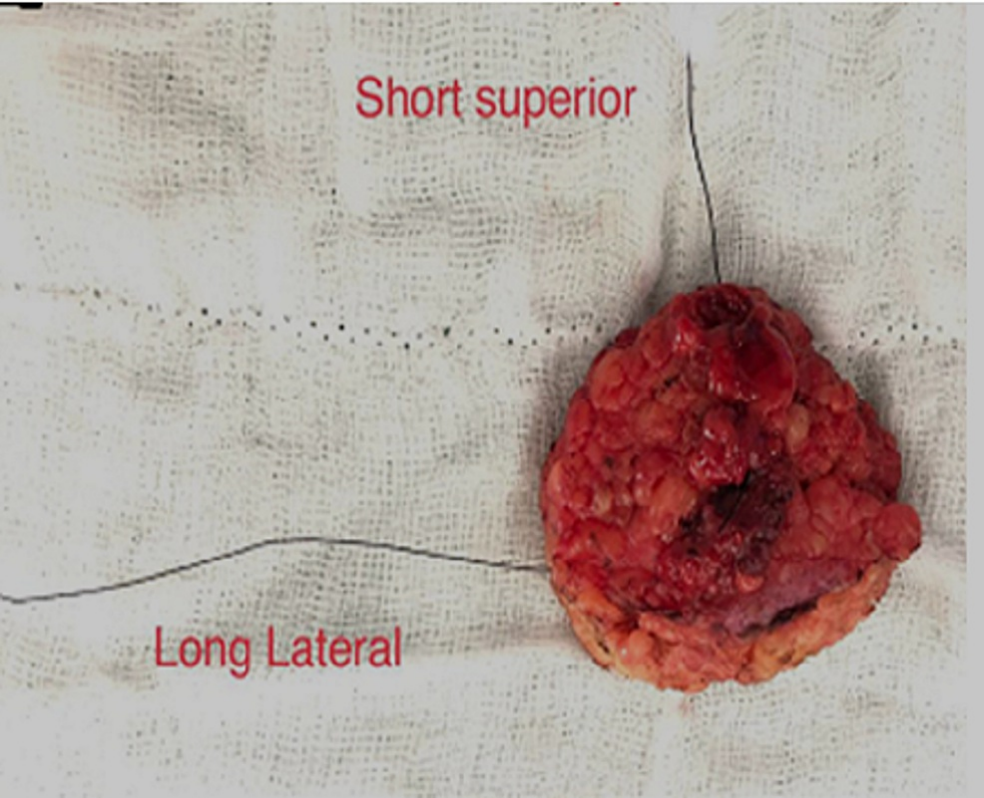
Specimen orientation.

**Figure 5:**
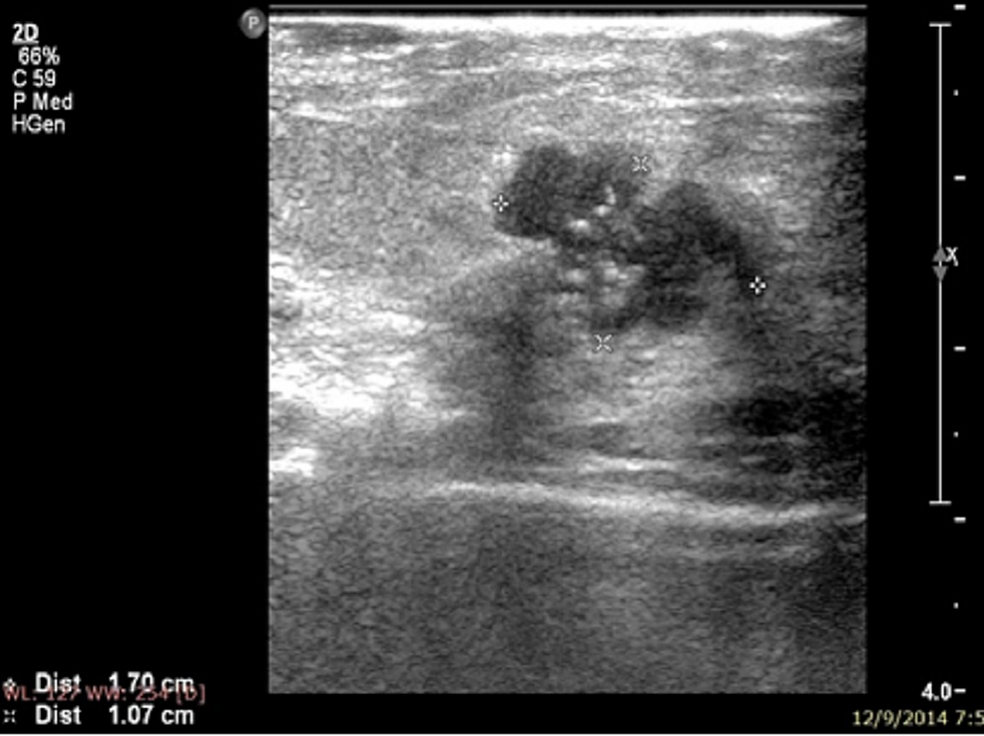
Specimen USG showing involved margins.

**Figure 6:**
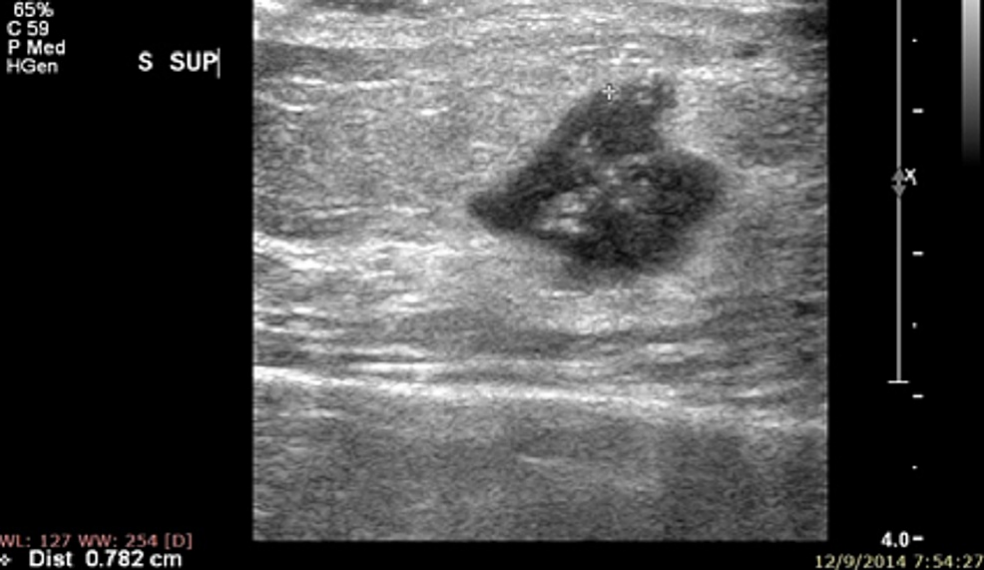
Specimen USG showing negative margins.

Specimen ultrasound (USG) was performed after orienting the specimen by the surgical team members well trained in breast ultrasonography. Specimens were oriented by the surgeon with sutures according to the convention “long lateral,” short superior,” and “double loop deep” (Figure 3, 4). The distance from the tumor edge to the surrounding margins was then measured by ultrasound and recorded for superior, lateral, inferior, medial, and deep resected margins. For each patient, we noted whether margins were free of tumor or not, (Figure 5, 6) whether margins were re-excised, whether these were the closest pathology margins and whether reoperation was required. The comparison was made between the margins commented by a pathologist in the final histopathology report and same assessed by ultrasonography. 

## Results

Sixty-two patients of early breast cancer, between the years 2013 to 2017, who underwent BCS were included in the study. The mean age of presentation was 45 years (range 28-68 years). Most patients had stage II disease (88%). Fifty-four percent of women were post-menopausal.

Performance of ultrasound and frozen section for assessing the margins

Specimen USG showed that the margins were positive in 16 cases (25.8%) and negative in 46 cases (74.2%). This correlated 100% with finding on final histopathology. Of these 16 cases, 10 (16.13%) were also positive on the frozen section. On table, re-excision of margins was done in all of these 16 cases and new margins were marked and sent for final histopathology. The histopathological examination confirmed the presence of disease (invasive or in-situ) in the margin shave sent separately in all of these 16 cases. The USG assessment was quite accurate and correlated well with findings on final histopathology. The specimen USG could accurately predict the margin status in 100% cases and had a sensitivity of 100%. Frozen section, however, failed to recognize the involved margin in six cases and an accuracy of 90.32%. The sensitivity of the frozen section for margin assessment was 62.5%, while that of USG was 100% (Table 1).

**Table 1:** Comparison between sensitivity and specificity of frozen section (Histopathology as gold standard)

	Sensitivity	Specificity	Negative likelihood Ratio	Positive Predictive Value	Negative Predictive Value	ACCURACY
SPECIMEN USG	100%	100%	0	100 %	100%	100%
FROZEN SECTION	62.50%	100%	0.38	100%	88.46%	90.32%

Further, In the intraoperative USG 16 cases were margin positive, in which re-excision was done in all and send to histopathology as a new margin. In these 16 new margins, 14 cases had clear margin and two had involved margin with ductal carcinoma in situ (DCIS) even after the second excision. Later these two opted for mastectomy. None of the cases had margin positivity on final histopathology that was not detected by USG. On comparing the area under the curve (AUC) of the frozen section and USG, AUC in the frozen section was 0.813 and USG was 1 (Table 2), which was significant and indicates specimen USG is superior in assessing margin than frozen section.

**Table 2:** Comparison of two ROC curves ROC: receiver operating characteristic

	AUC	SE	95% CI	Difference Between areas	95% Confidence interval	Significance Level
SPECIMEN FROZEN	0.813	0.0625	0.693-0.900	0.188	-0.0650 – 0.310	0.003
SPECIMEN USG	1	0	0.888 to 1.000			

## Discussion

Breast cancers are heterogeneous tumors imposing a challenge in treatment. It is the commonest cancer in women and is a leading cause of cancer mortality [1]. Large number of breast cancers in developing countries are locally advanced at presentation (30-60%) [2]. These cancers and a majority of them require NACT to become amenable for surgery. With increasing awareness and screening modalities, an increasing number of women are now detected in early stages and have a curable disease. BCS is a feasible and preferred option in many of these women because of improved cosmetic and psychological outcomes from breast conservation. The most important disadvantage of BCS is a lifelong risk for local recurrence, for which additional surgery may be required. The primary aim in BCS is to achieve a negative surgical margin while still preserving the aesthetically appealing breast. Adequate surgical margins have shown to decrease the incidence of local recurrence of the primary tumor [6-10]. To ensure negative margins, many techniques are suggested to assess the surgical margins. These include microscopic pathological studies, frozen section analysis, USG, and intraoperatively imprint cytology but with varying degrees of success [11,12]. In the current study, we evaluated the performance of specimen USG and the frozen sections as tools for assessing the margins.

Frozen section may be cost-effective, but is labour intensive and requires a dedicated pathologist. It may not be routinely available at most centers. There are technical challenges in freezing breast tissue, especially fat [13]. Fat are inaqueous materials need very low temperature in comparison to aqueous tissue for freezing and to make hard for better section. So, using the standard frozen section technique, it's difficult to section lumpectomy specimen with a high adipose component, leading to difficulty in the assessment of microscopic measurements. Another limitation of the frozen section is only a small fraction of the specimen margin is analyzed. Thus, a large percentage of breast tissue is not properly accessed in comparison of ultrasonography of resected tissue/specimen. The preoperative and intraoperative care process is less complex, which saves time and money. With intra-operative USG, the surgeon readily obtains the three-dimensional assessment of the tumor, achieving more accurate surgical planning [14,15]. US guidance also provides better operational anatomy coordination, which causes a surgeon to end up with a better clearance of margins and detect any multifocal disease missed during lumpectomy.

Ultrasound has been used for the intraoperative assessment of margins with standard clinical instrumentation and frequency ranges (7.5-14 MHz) [16-18]. Results show a significant reduction in positive margin resection rates from 41% to 9% [17] and 29% to 3.5% [16]. Recent studies showed that high-frequency ultrasound (20-80 MHz) may be able to differentiate between normal, benign, and malignant pathologies in resected margin specimens based on the spectral signatures in the ultrasonic signals [18,19].

In this study of 62 patients, the results are encouraging for the routine use of intra-operative ultrasound. It accurately assessed the margins in all cases in comparison to the frozen section, which assessed margins accurately in 62.5% of cases. Also, it appears from the observations and ROC curves that the area under the curve of frozen section was 0.87, whereas in the case of USG it was 1.0, which shows that intra-operative ultrasound assessment of the specimen can serve as a reliable and accurate replacement for frozen section.

## Conclusions

Accurate intra-operative assessment of margins has been an everlasting hunt in breast surgery. There are many methods used. From the present study, it could be inferred that intra-operative USG could accurately assess excision margins and can compliment frozen section, especially when breast conservation is offered to the patient, and has potential to diminish the number of subsequent undesired mastectomies for positive margin(s). Studies with a larger number of patients may be helpful for concretely establishing the role of USG for margin assessment in BCS as routine.

## References

[1] Momenimovahed Z, Salehiniya H (2019). Epidemiological characteristics of and risk factors for breast cancer in the world. Breast Cancer (Dove Med Press).

[2] Gedam MC, Shukla K, Ingale LY (2018). Clinical presentation and management of locally advanced breast carcinoma. Int Surg J.

[3] Fitzal F, Gnant M (2006). Breast conservation: evolution of surgical strategies. Breast J.

[4] (1990). Early stage breast cancer: consensus statement. NIH consensus development conference, June 18-21, 1990. NIH consensus development conference, June 18-21, 1990.

[5] McCahill LE, Single RM, Aiello Bowles EJ (2012). Variability in reexcision following breast conservation surgery. JAMA.

[6] Koppiker CB, Chintamani Chintamani, Dixit S (2019). Oncoplastic breast surgery in India: thinking globally, acting locally. Indian J Surg.

[7] Schwartz GF, Veronesi U, Clough KB (2006). Consensus conference on breast conservation. J Am Coll Surg.

[8] Cabioglu N, Hunt KK, Sahin AA (2007). Role for intraoperative margin assessment in patients undergoing breast-conserving surgery. Ann Surg Oncol.

[9] Dick AW, Sorbero MS, Ahrendt GM (2011). Comparative effectiveness of ductal carcinoma in situ management and the roles of margins and surgeons. J Natl Cancer Inst.

[10] Lombardi A, Pastore E, Maggi S (2019). Positive margins (R1) risk factors in breast cancer conservative surgery. Breast Cancer (Dove Med Press).

[11] Kaufman CS, Jacobson L, Bachman B, Kaufman L (2002). Intraoperative ultrasound facilitates surgery for early breast cancer. Ann Surg Oncol.

[12] Jin M, Kim JY, Kim TH, Kang DK, Han SH, Jung Y (2019). Intraoperative specimen mammography for margin assessment in breast-conserving surgery. J Breast Cancer.

[13] Maloney BW, McClatchy DM, Pogue BW, Paulsen KD, Wells WA, Barth RJ (2018). Review of methods for intraoperative margin detection for breast conserving surgery. J Biomed Opt.

[14] Ramos M, Díaz JC, Ramos T, Ruano R, Aparicio M, Sancho M, González-Orús JM. (2013). Ultrasound-guided excision combined with intraoperative assessment of gross macroscopic margins decreases the rate of reoperations for non-palpable invasive breast cancer. Breast.

[15] Zavagno G, Dona M, Orvieto E (2010). Separate cavity margins excision as a complement to conservative breast cancer surgery. Eur J Surg Oncol.

[16] Moore MM, Whitney LA, Cerilli L, Imbrie JZ, Bunch M, Simpson VB, Hanks JB (2001). Intraoperative ultrasound is associated with clear lumpectomy margins for palpable infiltrating ductal breast cancer. Ann Surg.

[17] Davis KM, Hsu C-H, Bouton ME, Wilhelmson KL, Komenaka IK (2011). Intraoperative ultrasound can decrease the re-excision lumpectomy rate in patients with palpable breast cancers. Am Surg.

[18] Doyle TE, Factor RE, Ellefson CL (2011). High-frequency ultrasound for intraoperative margin assessments in breast conservation surgery: a feasibility study. BMC cancer.

[19] Ouyang Y, Tsui PH, Wu S, Wu W, Zhou Z (2019). Classification of benign and malignant breast tumors using h-scan ultrasound imaging. Diagnostics.

